# A Review of the Field of Veterinary Science1Presented at the Thirty-fourth Annual Convention of the United States Veterinary Medical Association, Nashville, September, 1897.

**Published:** 1897-09

**Authors:** Leonard Pearson

**Affiliations:** Veterinary Department of the University of Pennsylvania


### A REVIEW OF THE FIELD OF VETERINARY SCIENCE.[1]

By Leonard Pearson, B.Sc.,V.M.D.,
VETERINARY DEPARTMENT OF THE UNIVERSITY OF PENNSYLVANIA.

It is well that we put aside our regular work from time to time and meet in this fashion, and upon such occasions it is appropriate for us to review the recent progress made by ourselves and by our profession. We can thus take our bearings and ascertain exactly where we stand in relation to the other professions, the commerce of the nations, and to our own past. There is also a temptation, particularly strong at such times, to peer into the future. So long as we confine ourselves to our regular work, to the plains and the valleys of things that are familiar to us, it is not possible to obtain this information nor to make these observations. It is only by comparing experiences, by adding the newly acquired knowledge of one member to that of another, by eradicating the ruts into which we fall so readily, and by resting our personal knowledge, thus improved and amplified, on the mountain composed of the accumulated wisdom of the ages, that we can obtain a complete view of our immediate surroundings and relations, and even an imperfect glimpse of the future.

In reviewing the field of veterinary science it is necessary to briefly consider the growth of this subject in order to fully realize our present station. The beginning of veterinary science coincides with the beginning of medicine, and is buried in the history of ancient Egypt and India. It is well known that the ancient Egyptians owned and cultivated domestic animals, especially the

[1] Presented at the Thirty-fourth Annual Convention of the United States Veterinary Medical Association, Nashville, September, 1897.

dog, ox, ass, goat, and goose. There is also evidence that the Egyptians bred antelopes in immense herds, and that this practice was given up some two thousand years before Christ, because other animals, particularly the sheep, displaced them. Although animals have been domesticated in all parts of the world, in more recent times the present list does not include more than twenty-five species, and has received no additions for several hundred years.

Animals constituted the principal wealth of the ancient roving tribes that covered Asia and gradually worked their way into Europe and Africa. It is but natural that when accident or disease overcame this valuable property that attempts should have been made to save it. Thus veterinary medicine began. As new views and theories arose in reference to the treatment of diseases of man these were applied without essential modification to the treatment of diseased animals; but the early assistance that general medicine derived from veterinary medicine is far greater than this, because custom did not permit the dissection of human bodies, and all anatomical knowledge was obtained by dissecting animals, and all knowledge of the processes of disease was derived from the same source.

As time passed more and more attention was devoted to the study of the diseases of man, and veterinary medicine was relegated to an inferior position. This tendency increased until, as a result of certain influences, domestic animals became valuable and indispensable when it became apparent that something should be done to develop means for controlling the diseases that swept them off by thousands, threatened the health and interfered with the prosperity of the people.

With the revival of learning in the sixteenth century, the study of veterinary medicine was taken up by many men of much ability and of high station, but these men were not skilled in the fundamental branches of medicine, and their labors consisted principally in rearranging and compiling the knowledge that had been acquired and recorded by their predecessors. In other words, veterinary medicine was not studied from a medical standpoint but from the standpoint of agriculture, and we find the writings on veterinary medicine included in larger works on rural affairs.

At that time there were many books on horsemanship, the breeding and general care of animals, and on animals in their relation to agriculture, but scientific treatises on veterinary medicine in any of its phases were lacking, with the single exception of the *Anatomy* of Carl Ruini. This book, issued in the year 1590, marks the beginning of scientific veterinary literature, but, strange to say, the other branches of our science developed so slowly that it was not until two hundred years later that works on the other fundamental branches equal to the *Anatomy* of Ruini were avaliable.

However, sufficient progress was made during this early period to indicate that the science was of value, and as changed conditions developed in the habits of the people, in agriculture, in transportation, and in warfare, the infectious diseases of animals became more widespread and caused more and more oppressive losses. The diseases that caused the greatest losses during the seventeenth and eighteenth centuries were anthrax, foot-and-mouth disease, lung-plague, rinderpest, glanders, influenza, distemper, mange, sheep-pox, and various helminthic diseases.

In the last century, while all of the communicable diseases prevailed more or less extensively, rinderpest was most prevalent and caused the greatest amount of distress and loss. It ravaged the southern provinces of Russia, Poland, many of the States of Germany, Hungary, Austria, Italy, France, and Switzerland. It was so virulent in Holland that more than 200,000 cattle were destroyed by it in 1714; in Italy 70,000 cattle died in one year. During the entire century the disease came and went in waves, certain years being marked by especially severe invasions. For a time it subsided in Holland to such an extent that the herds were re-established, but in the two years from 1744 to 1746 another serious invasion occurred, and once more 200,000 cattle were lost, thus impoverishing a large proportion of the population. A number of serious outbreaks also occurred in England. In 1747, 40,000 cattle were lost in Nottingham and Leicestershire, and 30,000 in Cheshire in six months, after which the progress of this plague was checked by the slaughter of about 80,000 animals. Foot-and-mouth disease prevailed extensively in France and other Continental countries, and anthrax rendered it impossible to rear live-stock in many localities in Europe. Glanders also prevailed very extensively in all parts of Europe.

It was about this time that the stage-coach became prominent, and vast numbers of horses were needed for this new work. There were also great numbers of large estates where many horses were bred, and the need of more knowledge and better skill in treating them became painfully apparent. The cavalry and artillery arms of the military service were also in a a state of rapid development, and veterinary knowledge was in great demand in this connection. The field was ripe for the establishment of a veterinary school, and when Bourgelat organized the first one at Lyons it was received gladly and recognized as a great advancement in all parts of Europe. The success of this institution was so great that students were sent to it by all of the principal rulers, and they in turn established veterinary schools in their own countries, all of them closely copying the French system.

While the success of these schools was very great in so far as obtaining students and government aid was concerned, the fact remains, and sad it is it must be stated, that the results of these schools for the first decades of their existence were most disappointing, and they did not succeed in drawing to them as students young men who were well trained and came from the better classes of society. While the pupils sent by foreign governments were of the better class, most of them being surgeons, skilled horsemen, and educated agriculturists, those that followed and constituted the great majority of the student body in all of the veterinary schools during the first years of their existence were from the lower classes, and it is reported that many of them were unable to read or write. The teachers in these schools during this period, which has very properly been termed the "Empirical Period" in veterinary education, were not well-trained veterinarians because there had been no opportunity for the development and acquisition of a thorough knowledge of veterinary science, and most of them were surgeons who had a certain fondness for animals, and who had amplified their knowledge of medicine by adding to it some of the empirical, unscientific, and in large measure worthless teachings of the existing literature. Of course, it could not be expected that the influence of the old empirical systems that had grown and developed for hundreds of years could be thrown off at once, and a scientific course in veterinary medicine could not be given before the science of veterinary medicine existed. It is, therefore, not surprising that the veterinary schools failed to accomplish the work that was expected of them, and succeeded only in turning out men who were not above the level of the best of the unskilled veterinarians who preceded them.

Another condition that has an important bearing upon our study of veterinary medicine was the change in the method of transportation that followed the introduction of steam locomotives. This led to a sudden and tremendous increase in domestic and foreign commerce, to greater activity in all kinds of business and the establishment of keener competition in rural affairs, and the development of new systems of husbandry. Under the old conditions, local wants were supplied entirely by local products, and what a locality did not produce was not consumed in that district. With the improvement in methods of transportation, moreover, the wants of the people increased at least as rapidly as the means for supplying them, and changes were seen not only in methods of conducting business, but also in the social life and especially in the food of the the people.

Lydten, in a scholarly paper read before the last International Congress at Bern, called attention to the influence of this development on the diet of the people, and has shown that while meat is now at least three times as expensive as it was sixty years ago, about three times as much per capita is consumed by the inhabitants of European countries. It seems to be well established that the degree of activity of a people is indicated by the amount of flesh consumed. A more than proportionate growth has occurred in the consumption of dairy-products. It is not necessary for one to be very old to remember when milk was a rarity and butter a luxury, and to recall how these articles of diet have displaced other substances made from fruits and vegetables. The fashion in clothing has also changed to such an eextent that far more wool is now required to properly clothe a person than was needed a comparatively few years ago.

All of this has led to an astonishing growth of the live-stock industry, so that the public is far more dependent upon it now than it was in the last century, when the first veterinary schools were established. The growth of veterinary science has been proportionate to the growth of the industry that is protected by it, and without such development in veterinary science the live-stock industry could not have attained its present proportions, and the progress of the whole people would have been restricted.

When it became evident in the earlier decades of veterinary education that the schools then in existence were not properly filling their functions, and after a sufficient number of well-trained veterinarians had developed, a thorough reorganization was accomplished for the purpose, as expressed in the report of the German Royal Commission: "To improve the present empirical material, and, in addition to the practical veterinarians that now exist, to educate scientific veterinarians who can be employed to direct veterinary police measures and to teach in veterinary schools." As a result of this change, which was made in all of the original schools, veterinary education became more scientific, the scope of the schools was enlarged, more and better teachers were appointed and the result has been gratifying in the highest degree. Fortunately, these changes were made in the earlier years of the present century, and their effect has been evident for more than two generations.

Let us consider for a moment what good has been accomplished by the veterinary schools and what effect their work has had on national life and prosperity. Undoubtedly, the most effective work has been in the direction of controlling the infectious diseases of animals. Rinderpest has been exterminated from Europe, and does not prevail anywhere extensively, excepting in South Africa, where it is now numbering its victims by thousands, and is interfering most seriously with the progress of the country. If South Africa belonged to a state of Continental Europe their efficient veterinary police measures would have been introduced long ago and the disease exterminated. Unfortunately, England does not avail herself of the highest veterinary skill, and thus there is no opportunity for the amazing results that have followed its application in Continental countries.

Lung-plague, or contagious pleuro-pneumonia, has been controlled to such an extent in Europe that it does not cause serious losses at this time. Notwithstanding the fact that it once secured quite a foothold in this country, modern methods, as administered originally by Drs. Law, Thayer, and Lyman, and later by Dr. Salmon, have succeeded in entirely eradicating it. When we remember that pleuro-pneumonia was especially virulent in this country, due to the new soil that it found to operate on, to the fact that this is essentially an agricultural and cattle-breeding land, the immense scope of our territory, and the foothold that the disease had gained, we can justly consider its extermination as one of the greatest triumphs that veterinary science has achieved at any time in any country. If the disease had been dealt with as it was in England at that time, it would have spread over the entire face of the country and caused losses amounting to so many millions of dollars that it is impossible to estimate them.

Anthrax has been studied with such success that an efficient vaccine has been discovered by means of which animals can be inoculated and the development of the disease prevented. Moreover, so much light has been thrown on the life-history of the germ that we now know what measures must be taken to prevent the spread of this disease or its establishment in a certain locality after it has been introduced.

Foot-and-mouth disease, although it still occurs in most of the Continental countries, has succumbed to the veterinary police measures that are enforced to such an extent that it has not caused serious loss for a considerable number of years.

Mange of horses and scab of sheep, diseases that were formerly dreaded so acutely by horsemen and shepherds, are now comparatively rare. Distemper and glanders of horses occur from time to time, but they are kept in check so well that they no longer seriously menace the horse-breeding industry.

Texas fever, a disease which formerly destroyed thousands upon thousands of cattle every year in the United States, has been studied so thoroughly by Drs. Theobald Smith, F. L. Kilbourne, and others, under the auspices of the Bureau of Animal Industry, that preventive measures based on their discoveries are so successful that outbreaks of Texas fever are practically unknown north of the "Texas Fever Line." A few years ago outbreaks of Texas fever were very common; they occurred in the most unexpected localities and destroyed so many cattle that drovers and farmers were kept in a state of constant apprehension. Last year there was but one small outbreak in Pennsylania, and this year not a single case has been reported in that State. This, also, is one of the greatest triumphs of veterinary medicine. But development in this direction is not complete, and the researches that are now in progress will, it is hoped, lead to some effective measures under which Northern cattle can be introduced with safety into Southern States and Southern cattle can be brought north at any season.

Hog-cholera is a disease upon which a good deal of light has been shed during the past few years, but we are still without an effective means of preventing its ravages.

Tuberculosis is an affection that is arousing much discussion at this time, is occasioning most extensive losses, and must be dealt with more seriously in the near future.

The immense field opened by the discoveries in serum-therapy is of equal interest and importance to the physician and veterinarian, and much of this recent development results from the work of distinguished members of our profession.

The international trade in live-stock has developed enormously during the past few years. It is a comparatively small matter to ship cattle three thousand, four thousand, or five thousand miles, and with modern facilities these journeys can be made in a very few days. International trade in live-stock and live-stock products, such as hides, skins, and wool, has also reached immense proportions. These also are carried quickly between points on opposite sides of the world.

It will thus be seen that without proper supervision it would not be difficult for the most dangerous diseases to be carried long distances from the most remote countries into previously uninfected territory. But every civilized country has a force of efficient veterinarians and a more or less perfect system of quarantine and control, by means of which the ravages of disease that would otherwise be conveyed by this international commerce are avoided. It is appalling to consider the effect upon the nations of the abandonment of these systems. The United States would become reinfected with lung-plague, and we would quickly import the foot-and-mouth disease, sheep-pox, swine erysipelas, dourine, and possibly rinderpest and other deadly affections. Our live-stock industry represents an investment at this time of about two billion dollars. A few of these diseases, if uncontrolled by the application of modern veterinary measures, would quickly devastate it to the amount of 25 per cent. at least. This means a loss in cash value of $500,000,000, and there is no telling where the calamity would stop. On account of our somewhat isolated position we can protect ourselves better from the invasion of infectious diseases than the countries of Europe. There it is necessary to keep up a constant control of the most elaborate type, but it is successful in protecting the vast wealth invested in live-stock and in perpetuating the most important food-supply of the country.

The *indirect* importance to agriculture of protecting our animals is made apparent when we consider the value of their manure and attempt to imagine the appearance of our fields and farms unfed, unrenovated by this plant-food. While some districts could subsist for a time under these conditions, the vast majority of our farms would gradually lose their fertility, and become barren wastes.

In addition to the protection of the live-stock industry as a whole, which, it is evident, is essential to the continued prosperity of the country, the veterinarian finds important and remunerative occupation in the treatment of individual animals. As veterinarians become more proficient it is becoming more and more evident to the owners of live-stock that it is not profitable to allow their animals when ill or injured to languish or die or make such imperfect recoveries that a large portion of their value is destroyed, when many of these losses can be prevented by the employment of suitable veterinary skill. The veterinarian not only enables the live-stock owner to save money by preserving the life of his animal and restoring it to usefulness, but it is also economical to know that animals in certain conditions cannot recover. They can, then, upon the advice of the veterinarian, be destroyed painlessly, and the owner avoids the purchase of special food, medicine, and appliances and many days of expensive attention.

In its relation to public health, veterinary science is constantly growing in importance. It is well known that a number of diseases to which people are liable can be conveyed by flesh and milk, and the long-continued experiences of several of the most highly civilized European countries teaches us that these important foods cannot be properly guarded and their healthfulness assured without the employment of veterinarians to examine, inspect, and advise in reference to the management of the animals that produced them, the conduct of the dairy, the character of the flesh, as determined by post-mortem examination, and the care of these delicate foods until they are consumed. In Germany nearly every city has a municipal abattoir wherein all cattle killed for local consumption must be dressed under veterinary supervision. Each abattoir is under the management of a veterinarian with the title of director, who performs all the inspections himself, or, if the business is too great for this, is assisted by as many veterinarians as may be needed. In Berlin about one hundred are engaged in this work. The assistants are not, however, continuously employed. They are in most cases local practitioners, who are engaged to devote certain hours each week to this work, and are paid in proportion to the time occupied. In this country the National Government, through the Bureau of Animal Industry, finds it necessary to employ a large number of veterinarians in work of a similar character.

The relation of the veterinarian to the improvement of live-stock, through the introduction of improved breeds and species, and improvement by care and selection is a very important one, but one that has not received sufficient attention in this country. While there is a high general adaptability to their special purposes of the various species and varieties of domestic animals that are used in the United States there is still room for great improvement, and the average individual efficiency should be increased from 25 to 50 per cent. This can only be affected by a more thorough understanding and general application of the principles of breeding, selection, and heredity; by the education of breeders to recognize and more fully appreciate the valuable points and the defects of animals kept for different purposes; by the better feeding and care of animals during the formative period, and by a closer observance of the laws of hygiene and sanitation. Much can be accomplished through agricultural fairs, and exhibitions and live-stock shows.

Every veterinarian who has visited many exhibitions can recall instances of a most dangerous and destructive character wherein animals with hereditary unsoundness and defects of conformation have been awarded premiums. It is the duty of every veterinarian to thoroughly post himself on all branches of zoötechnics, on breeding problems, and general live-stock affairs, so that he may be qualified to serve in an advisory or executive capacity at live-stock exhibitions.

In view of the past achievements of veterinary science and the local, national, and international importance of its work at this time, there can be no question as to the future of this profession. In order, however, that the highest development may be obtained it is necessary that we should grasp the full significance of our labors. The trained veterinarian is naturally the expert on all live-stock questions, and upon him devolves the responsibility for the improvement and extension of this foundation branch of commerce and national prosperity. When viewed in these enlarged relations, there is more need for veterinary knowledge now than ever before; but to satisfactorily fulfil all of these modern requirements it is necessary that the veterinary students should receive more special training in some subjects that are now looked upon as minor branches by most of the veterinary schools.

When the veterinarian is the generally acknowledged expert on all questions of animal husbandry, and is freely consulted in matters of hygiene, breeding, selection, and feeding, the construction of stables and related matters, as well as on pathological questions, his field of usefulness and occupation will have become so broad that there will be a far greater demand for his services in every breeding and farming district, and this will continue to grow as the direct advantages afforded by such consultations become evident.

